# Reliability, Quality, and Educational Suitability of TikTok Videos as a Source of Information about Scoliosis Exercises: A Cross-Sectional Study

**DOI:** 10.3390/healthcare10091622

**Published:** 2022-08-25

**Authors:** Chan Woong Jang, Myungsang Kim, Seong-Woong Kang, Han Eol Cho

**Affiliations:** Department of Rehabilitation Medicine, Gangnam Severance Hospital, Rehabilitation Institute of Neuromuscular Disease, Yonsei University College of Medicine, Seoul 03722, Korea

**Keywords:** education, exercise, idiopathic scoliosis, PSSE, Tiktok

## Abstract

This study aimed to systematically assess the informational reliability, quality, and educational suitability of videos introducing scoliosis exercises on TikTok. We retrieved and screened 1904 TikTok videos with the hashtags: “#scoliosis”, “#scoliosisexercise”, and “#scoliosistips”, before collecting a final sample of 171 scoliosis exercises in March 2022. Then, two independent raters assessed the reliability and quality of the videos using the DISCERN instrument and evaluated the educational suitability of the information using “Scoliosis Exercise Education Score” (SEES; exercise cycle, target, effect, precaution, and rationale). None of the videos were rated as excellent or good according to DISCERN. The mean SEES score was 2.02 out of 5. Videos uploaded by health organizations had significantly lower DISCERN and SEES scores than those by general users and healthcare professionals. Regarding the propriety of physiotherapeutic scoliosis-specific exercises (PSSE), DISCERN and SEES scores were significantly higher in the PSSE proper group than in the PSSE non-proper group. Although TikTok has become a popular source of scoliosis-related information, the overall information quality, reliability, and educational suitability of videos on scoliosis exercises in TikTok appear to be low, suggesting that TikTok is not suitable source for obtaining scoliosis exercise information.

## 1. Introduction

Idiopathic scoliosis is defined as an abnormal curvature of the spine with a radiological lateral Cobb angle of ≥10°, which occurs mainly in children and adolescents without underlying congenital or neuromuscular abnormalities [1,2]. It is the most common form of scoliosis, and epidemiological studies estimate that about 1–4% of adolescents experience some degree of spinal curvature [3]. If scoliosis exceeds a critical threshold, usually considered to be a Cobb angle ≥30°, there are higher risks of health problems in adult life, decreased quality of life, cosmetic deformity, visible disability, pain, and progressive functional limitations [4,5,6]. Adolescence is a critical period in which scoliosis can be prevented [7]. Current evidence suggests that conservative scoliosis treatment is effective at stopping curve progression and improving the curves at skeletal maturity [8]. Bracing and physiotherapy scoliosis-specific exercise (PSSE) are the two main conservative treatments [8]. Usually, patients with a Cobb angle of up to 25° receive exercises alone, and those with a Cobb angle between 25° and 50° receive additional bracing [9]. A previous long-term randomized clinical trial study found that PSSE improved skeletal maturity by decreasing the Cobb angle in patients with adolescent idiopathic scoliosis [9].

Emerging technologies provide great health communication opportunities, particularly through video-based platforms [10,11,12,13,14]. TikTok is an online video platform where users can post a short, 30 s video. It was released in 2017 and has garnered over a billion users worldwide in just a couple of years [15]. It has attracted significant research attention owing to its influence on health communication. Many studies have performed TikTok video analysis for various diseases, such as coronavirus disease 2019 (COVID-19), diabetes, and chronic obstructive pulmonary disease (COPD) [16,17,18].

TikTok can be an important source of information about idiopathic scoliosis management, considering that 41% of TikTok users are between the ages of 16 and 24, together with the ease of sharing information on the platform [19,20]. Although several videos about scoliosis exercises can be found on TikTok, the information quality they offer remains unclear. Hence, this study aimed to systematically assess the information reliability, quality, and educational suitability of videos on scoliosis exercises on TikTok.

## 2. Materials and Methods

### 2.1. Search Strategy and Data Collection

**Figure 1:**
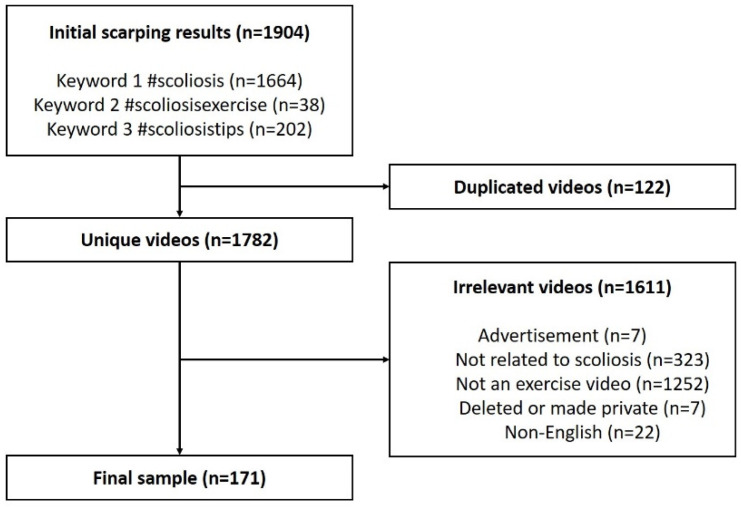
Flowchart of the search process for videos related to scoliosis exercises.

A cross-sectional TikTok search was conducted via Python crawling to retrieve the videos related to scoliosis exercises on 2 March 2022, with the following three hashtags: “#scoliosis”, “#scoliosisexercise”, and “#scoliosistips”. Initially, we searched with “#scoliosis”, but not enough exercises videos were returned. After conducting several pilot searches using various combination of hashtag words and expressions, we consensually chose the three hashtags. All searched videos (*n* = 1904) underwent a selection process to select the most relevant ones. We excluded videos that were (1) duplicated (*n* = 7), (2) irrelevant to scoliosis (*n* = 323), (3) not directly related to scoliosis exercises (*n* = 1252), (4) inaccessible because of deletion or privacy at the time of analysis or privacy (*n* = 7), and (5) in non-English languages (*n* = 22). Finally, 171 videos remained for data analysis (Figure 1).

The metadata collected for each video included users’ data (uploader’s ID, number of followers, and followings of the uploader) and videos data (description of the video, uniform resource locator, upload date, posting days, duration in seconds, number of views, likes, shares, comments, and text of the video). In addition, based on the method of previous studies, the viewing index (number of views/posting days) and engagement index (number of likes, shares, and comments) were calculated [21,22]. As this study did not involve any human participants or animals, ethics committee approval was not required.

### 2.2. Scoring Systems

We employed two scoring systems for assessing videos: DISCERN for assessing reliability and quality, and the ‘Scoliosis Exercise Education Score (SEES) to evaluate the educational suitability of the information in each video.

#### 2.2.1. DISCERN for Reliability and Quality Assessment

DISCERN, developed by Charnock et al. comprises 16 questions: the first eight questions concern the reliability of the publication, the next seven reflect the quality of the publication’s information, and the last question rates the overall quality as a source of information [23]. DISCERN has been widely used and proven to be useful to assess the reliability and quality of written patient health information regarding treatment choices on other video-based platforms [24]. Considering the total average scores, the DISCERN results were categorized as follows: “excellent” (63–75), “good” (51–62), “fair” (39–50), “poor” (27–38), and “very poor” (15–26) [25].

#### 2.2.2. SEES for Educational Suitability Assessment

To evaluate the content of the exercise in detail, we developed the SEES. This instrument examines how accurately viewers could understand and follow exercises after watching the videos. It consists of the following five items: “Exercise cycle (does the video describe the exercise cycle?)”, “Target (does the video describe the target area of the exercise?)”, “Effect (does the video describe the expected effect of the exercise?)”, “Precaution (does the video describe the precautions of the exercise?)”, and “Rationale (does the video explain the rationale of the exercise?)”. The SEES calculation method is based on the sum of the points from the individual items. Each video obtained a score between 0 and 5. A higher score indicates more suitable information provided for educational purposes.

#### 2.2.3. Assessment

All videos were independently evaluated by two reviewers (M.K. and C.W.J.), who are certified physicians specialized in physical medicine and rehabilitation. After confirming that the metadata collected by the Python crawling were correct, we assessed the video content using the DISCERN and SEES. This process was performed after the two reviewers were trained to evaluate the videos in the same manner. Any discrepancies between the reviewers were resolved by discussion with the third author (H.E.C.), who is also a specialist in physical medicine and rehabilitation, and has sufficient experience with patients with scoliosis.

Furthermore, the included videos were categorized by uploader type into three groups: general users, healthcare professionals, and health organizations. The healthcare professionals group included individuals who described themselves as healthcare professionals, such as nurses, physicians, surgeons, physical therapists, or chiropractors. The health organizations group included hospitals, treatment centers, or for-profit organizations. There were no videos from non-profit organizations or news agencies. Then, the two reviewers (C.W.J. and H.E.C) with experience in PSSE as rehabilitation specialists divided the videos into two groups: those which provided exercise treatment that was appropriate for PSSE and those that did not [26]. When different grouping was obtained for the same video, the discrepancy was resolved by a third author (S.W.K.).

#### 2.2.4. Statistical Analysis

Descriptive data are presented as the mean (standard deviation (SD)), median (interquartile range (IQR)), and number (percentage). The Shapiro–Wilk normality test was adopted to evaluate data normality. One-way analysis of variance and the Kruskal–Wallis rank sum test were used to compare continuous and categorical variables, respectively. Multiple comparisons among the groups were also performed. Inter-rater reliability was measured separately for the scoring of DISCERN and SEES using Cohen’s weighted kappa coefficient, with significance set to *p* > 0.6. All analyses were performed using RStudio software (R version 4.1.2). Statistical significance was set to *p* < 0.05, for parameters other than inter-rater reliability.

## 3. Results

### 3.1. Basic Characteristics

Of all 1904 videos, 1664 were searched using #scoliosis, 38 videos using #scoliosisexercise, and 202 using #scoliosistips. After applying these exclusion criteria, 171 videos were included in the final analysis.

Table 1 presents the videos’ basic characteristics. The total number of views of the videos was 36,855,244. The median was 13,400.0 (IQR = 727.0–103,500.0). The median number of posting days was 246.0 (IQR = 97.0–441.0); thus, the median viewing index was 53.6 (IQR 5.6–319.1). The videos collectively received 2,989,661 likes, 23,863 comments, and 85,253 shares: medians of 1024.0 (IQR = 0.0–5225.0), 1024.0 (IQR = 0.0–5225.0) and 16.0 (IQR = 0.0–253.0), respectively. The median engagement index of the videos was 1057.0 (IQR = 18.0–5436.0). Average DISCERN and SEES scores of the included videos were 33.60 (8.22) and 2.02 (1.69), respectively. Among the subdomains of DISCERN, the quality of treatment choices domain scored the lowest (mean 1.65, SD = 1.01). According to the DISCERN results, none of the videos were rated as excellent or good. The kappa scores indicated good agreement between the raters, showing that the inter-rater reliabilities for DISCERN and SEES were 0.87 and 0.84, respectively.

### 3.2. Types of Uploaders

Table 2 presents characteristics according to the type of uploader. The results suggest that general users uploaded most of the videos (*n* = 92, 54.4%), followed by health organizations (*n* = 42, 24.6%), and healthcare professionals (*n* = 36, 21.1%). The viewing index and engagement index were the highest for healthcare professionals, followed by general users, and lowest for health organizations (*p* = 0.002, both).

**Figure 2:**
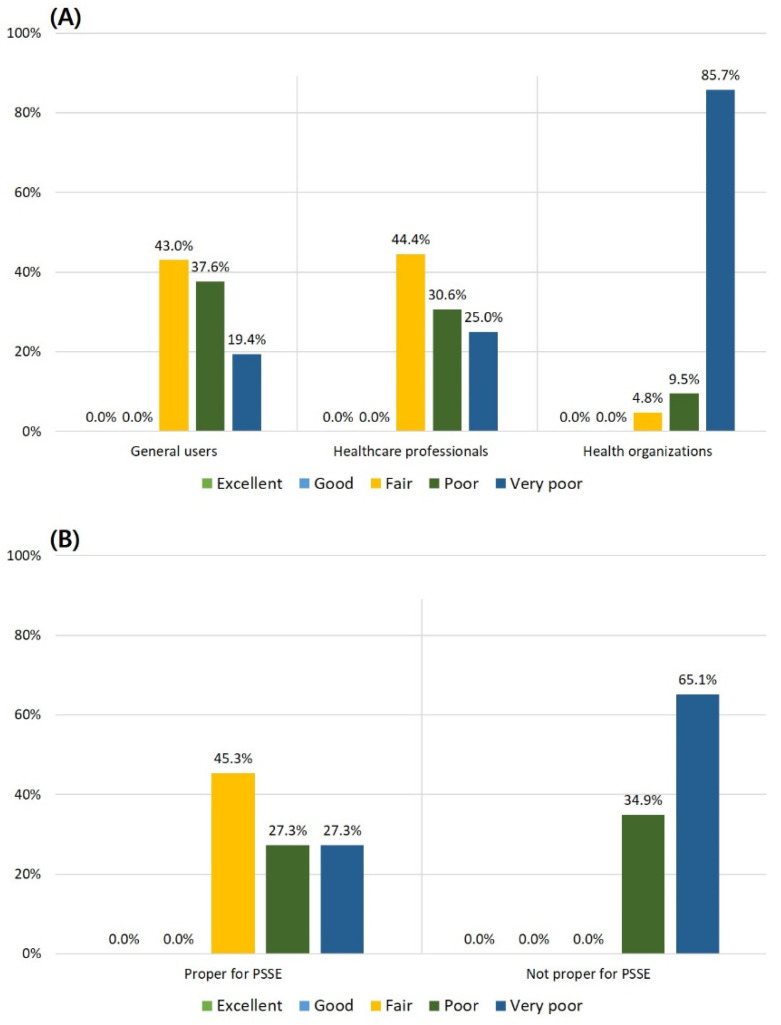
(**A**) Distribution of DISCERN grades across the type of uploader of included videos and (**B**) distribution of DISCERN grades across the propriety for PSSE.

The differences in the DISCERN score and the three subdomains (reliability of the videos, quality of treatment choices, and overall information quality) among the three types of uploaders were statistically significant (*p* < 0.001, *p* < 0.001, *p* = 0.003, and *p* < 0.001, respectively). Post-hoc analysis of the DISCERN scores revealed that videos uploaded by health organizations had significantly lower scores for DISCERN and the three subdomains than those uploaded by general users and healthcare professionals. Regarding the distribution of the DISCERN grades, there was no statistically significant difference in the proportion of poor or very poor grades regarding videos of general users (57.0%) and healthcare professionals (55.6%); however, for health organizations, the proportion was significantly higher (95.2%). The distribution of the DISCERN results of the videos according to the uploader type is shown in Figure 2A.

Regarding the SEES, there was no statistically significant difference between general users and medical professionals at 2.35 and 2.14 points on a 5-point scale, respectively (*p* = 0.628). However, health organizations scored significantly lower (1.19 points) than general users (*p* < 0.001) and medical professionals (*p* = 0.012).

### 3.3. Propriety for PSSE

Of the 171 images, 128 were PSSE appropriate, whereas 43 were not (Table 3). The viewing index was significantly higher in cases not suitable for PSSE (median 21.8, IQR 3.9–174.8) than in the videos appropriate for PSSE (median 274.3, IQR 58.8–1112.9) (*p* = 0.017). The engagement index was also significantly higher in cases not suitable for PSSE (median 92.0, IQR 12.3–3762.0) compared with the appropriate videos (median 4263.0, IQR 2075.0–14,117.0) (*p* = 0.012).

Regarding the evaluation of video content according to PSSE propriety, the DISCERN score was 35.98 ± 8.13 on average in PSSE appropriate videos, which was higher than the 26.49 ± 2.05 on average for the videos not appropriate for PSSE (*p* < 0.001) (Table 3). The reliability of the videos, quality of treatment choices, and overall information quality were all high (*p* < 0.005). The proportion of poor to very poor grades for the entire video showed a significant difference: 54.7% for appropriate PSSE videos and 100% for the inappropriate group (Figure 2B). SEES also showed averages of 2.63 and 0.21 points out of 5 for PSSE appropriate and inappropriate videos, respectively (*p* = 0.001).

## 4. Discussion

Prior to the wide spread of the Internet, patients could obtain medical information in limited ways, such as through face-to-face discussions with physicians. Currently, a variety of social media platforms have opened new horizons for patients to obtain medical information conveniently [27,28,29]. With the various strengths of social media, such as easy accessibility and worldwide influence, there is a positive expectation that these platforms will deliver medical information to patients easily, and ultimately establish a patient-centered medical environment [30]. However, there are concerns that information transmitted via social media will be biased and of low quality, resulting in erroneous information being provided to patients [31]. Although many researchers have conducted studies on medical information on social media, previous studies have mainly focused on YouTube. Since TikTok has emerged as a popular social media platform, we attempted to evaluate the content and quality of TikTok videos as an information source for scoliosis exercises.

Previous studies have shown that TikTok has great influence as a medium for health information. For example, COVID-19 related videos in TikTok were viewed approximately 93.1 billion times [17]. Videos related to COPD received 1.7 million likes and 175 thousand comments [18]. Videos about diabetes received 2.75 million likes and 157 thousand comments, and were shared 305 thousand times [16]. Our results are consistent with those of previous studies. We identified 171 videos on scoliosis exercises with 36 million views, 85 thousand shares, 3 million likes, and 23 thousand comments. In other words, TikTok is a powerful platform for providing information about scoliosis exercises.

However, videos of scoliosis exercises in TikTok have low reliability and quality. In general, the overall DISCERN score for 171 videos was poor (average score of 33.60). There were no videos that were categorized as “excellent” or “good”, and over 66% of the videos were ranked as “poor” or “very poor”. In particular, quality score was particularly low. In addition, the videos were not educationally suitable; all areas were explained in less than 50% of the total videos. In particular, very few videos explained precautions and exercise cycles. These poor results might be due to the limitation of TikTok itself; it can only contain brief content because the maximum playtime of a TikTok video is very short. The median length of the 171 videos included in our study was only 19.00 s, and the average was 24.29 s. In other words, TikTok is not a suitable platform for teaching scoliosis exercises. This result is different from previous studies conducted in China showing that TikTok is useful as a health information source for other diseases; diabetes-related videos showed a total DISCERN score of 40.00–50.64 [16] and COPD of 56.0–66.8 [18]. These discrepancies may be attributed to differences in diseases or the simplicity of the content covered. A previous YouTube analysis of kyphosis and scoliosis also found that videos had low quality, supporting our results [32,33].

Our study found that the informational reliability and quality in videos differed according to the type of uploader. Videos from general users and healthcare professionals did not show statistical differences, despite the videos of healthcare professionals receiving the most likes and comments and being most frequently shared by users. Interestingly, the videos published by health organizations had the lowest quality. We suspect that this is because a few health organizations uploaded many low-quality videos. Two institutions (@mobilitymedclinic/@scolidocs) uploaded 27 and 9 videos, respectively, accounting for 83.7% of all healthcare organization videos. However, the average DISCERN scores were 25.2 and 28.0, and the suitability scores were 0.71 and 0.5, respectively, which are much lower than the overall averages.

Our sample showed that PSSE appropriate videos have good reliability, quality, and educational suitability. This suggests that a higher level of expertise can be obtained by following the PSSE, an evidence-based exercise. Unfortunately, PSSE-based videos received fewer likes and comments and were less frequently shared by users, indicating that the objective quality of the information did not lead to credibility. Notably, most videos regarding PSSE contained only one exercise method. The PSSE includes various correction methods, such as 3D correction, breathing mechanics, muscle activation (stabilization), mobilization, and ADL change. These correction methods should be personalized and comprehensively applied. In other words, viewers may find it difficult to obtain sufficient scoliosis correction by following each TikTok video.

### Limitations

This study had several limitations. First, there may have been a selection bias depending on the search terms. We attempted to address this by selecting the most commonly used hashtags and analyzing all the videos, not just the most popular videos. Second, all videos were analyzed by two independent PMR specialists, and it is possible that subjective bias may have occurred during this process. To complement this, each independently analyzed and compared, and the third author went through the process of adjusting the discrepancies. Third, we did not use additional health information quality evaluation tools, such as the JAMA benchmark or HONcode, besides DISCERN. Although DISCERN has been proven effective for assessing the quality of videos on other platforms, future studies using additional tools are encouraged.

## 5. Conclusions

TikTok has become a popular source of information where people can easily gain access to health-related information about scoliosis. However, the reliability, quality, and educational suitability of videos about scoliosis exercise in TikTok were low, which suggests that the platform is not a suitable source of scoliosis exercise information. Medical professionals who specialize in the treatment of scoliosis should focus more on creating informative, trustworthy videos with education in mind while staying up to date on internet content to avoid viewers receiving false information. Additional research is also required to identify the best way to deliver validated scoliosis exercise methods to the general public.

## Figures and Tables

**Table 1:** Characteristics of included videos.

Characteristics, Median (IQR)	Total (*n* = 171)
Viewing index ^1^	53.6 (5.6–319.1)
Number of views	13,400.0 (727.0–103,500.0)
Posting days	246.0 (97.0–441.0)
Engagement index ^2^	1057.0 (18.0–5436.0)
Number of likes	1024.0 (0.0–5225.0)
Number of comments	1024.0 (0.0–5225.0)
Number of shares	16.0 (0.0–253.0)
Duration (seconds)	19.0 (12.0–31.0)
**Scoring, Mean (SD)**	
DISCERN	33.60 (8.22)
Reliability of the videos (item 1–8)	2.50 (1.33)
Quality of treatment choices (item 9–15)	1.65 (1.01)
Overall information quality (item 16)	2.06 (1.23)
SEES ^3^	2.02 (1.69)
Exercise cycle	0.15 (0.35)
Target area	0.40 (0.49)
Effect	0.42 (0.49)
Precaution	0.08 (0.27)
Rationale	0.23 (0.42)

^1^ Number of views/posting days. ^2^ Number of likes + comments + shares. ^3^ Shoulder exercise education scores.

**Table 2:** Characteristics of the videos by type of uploader.

Characteristics, Median (IQR)	Type of Uploader			*p* Value
	General Users ^1^ (*n* = 93)	Healthcare Professionals ^2^ (*n* = 36)	Health Organizations ^3^ (*n* = 42)	
Viewing index ^4^	43.8 (2.2–207.4)	500.6 (31.1–1976.7)	17.6 (5.6–186.1)	0.002
Number of views	25,200.0 (308.0–78,150.0)	118,750.0 (9948.5–357,175.0)	2061.5 (954.8–20,775.0)	0.003
Posting days	313.0 (98.5–525.5)	250.0 (91.5–445.5)	155.5 (72.8–256.5)	0.003
Engagement index ^5^	1716.0 (12.5–4964.0)	4116.0 (485.5–16,107.5)	37.5 (18.0–629.8)	0.002
Number of likes	1565.0 (11.5–4494.5)	3746.0 (364.3–15,700.0)	36.0 (18.0–569.0)	0.003
Number of comments	11.0 (1.0–47.0)	60.0 (11.8–136.5)	0.0 (0.0–7.0)	0.062
Number of shares	15.0 (0.0–130.5)	265.0 (39.5–671.5)	1.5 (0.0–34.0)	0.029
Duration (seconds)	20.0 (13.0–30.5)	21.5 (12.0–43.3)	15.0 (10.0–20.0)	0.054
**Scoring, Mean (SD)**				
DISCERN	35.69 (7.92)	36.06 (8.37)	26.86 (4.16)/28.74 (5.69)	<0.001
Reliability of the videos (item 1–8)	2.65 (1.37)	2.66 (1.41)	2.02 (1.05)/2.2 (1.17)	<0.001
Quality of treatment choices (item 9–15)	1.74 (1.10)	1.76 (1.09)	1.36 (0.57)/1.41 (0.68)	0.003
Overall information quality (item 16)	2.32 (1.23)	2.44 (1.32)	1.14 (0.47)/1.26 (0.65)	<0.001
SEES ^6^	1.65 (1.55)	1.47 (1.36)	0.29 (0.71)	0.005
Exercise cycle	0.18 (0.39)	0.19 (0.40)	0.02 (0.15)/0.05 (0.23)	0.036
Target area	0.53 (0.50)	0.44 (0.50)	0.10 (0.30)/0.21 (0.42)	<0.001
Effect	0.52 (0.50)	0.47 (0.51)	0.14 (0.35)/0.26 (0.45)	<0.001
Precaution	0.12 (0.32)	0.08 (0.28)	0.00 (0.00)/0 (0)	0.069
Rationale	0.30 (0.46)	0.28 (0.45)	0.02 (0.15)/0.05 (0.23)	0.001

^1^ Common TikTok users. ^2^ Individuals who describe themselves as healthcare professionals (physicians, physiotherapists.) ^3^ For-profit or nonprofit organizations or news agencies. ^4^ Number of views/posting days. ^5^ Number of likes + comments + shares. ^6^ Shoulder exercise education scores.

**Table 3:** Characteristics of the videos across propriety for PSSE ^1^.

Characteristics, Median (IQR)	Propriety for PSSE		*p* Value
	Proper for PSSE (*n* = 128)	Not Proper for PSSE (*n* = 43)	
Viewing index ^2^	21.8 (3.9–174.8)	274.3 (58.8–1112.9)	0.017
Number of views	3456.0 (365.5–79,850.0)	76,800.0 (28,100.0–282,700.0)	0.012
Posting days	189.5 (94.0–442.5)	317.0 (217.0–433.0)	0.019
Engagement index ^3^	92.0 (12.3–3762.0)	4263.0 (2075.0–14,117.0)	0.011
Number of likes	90.5 (11.3–3391.0)	4129.0 (2029.0–13,500.0)	0.007
Number of comments	2.0 (0.0–38.8)	56.0 (16.0–175.0)	0.004
Number of shares	5.0 (0.0–225.8)	35.0 (5.0–254.0)	0.026
Duration (seconds)	18.0 (12.0–26.8)	22.0 (12.0–40.0)	0.069
**Scoring, Mean ± SD**			
DISCERN	35.98 (8.13)	26.49 (2.05)	<0.001
Reliability of the videos (item 1–8)	2.65 (1.40)	2.03 (0.99)	0.003
Quality of treatment choices (item 9–15)	1.77 (1.11)	1.31 (0.47)	<0.001
Overall information quality (item 16)	2.40 (1.24)	1.05 (0.21)	<0.001
SEES ^4^	2.63 (1.49)	0.21 (0.56)	0.001
Exercise cycle	0.19 (0.39)	0.02 (0.15)	0.009
Target area	0.52 (0.50)	0.07 (0.26)	0.003
Effect	0.54 (0.50)	0.05 (0.21)	<0.001
Precaution	0.10 (0.30)	0.02 (0.15)	0.107
Rationale	0.29 (0.46)	0.05 (0.21)	0.001

^1^ Physiotherapy scoliosis-specific exercises. ^2^ Number of views/posting days. ^3^ Number of likes + comments + shares. ^4^ Shoulder exercise education scores.

## Data Availability

Data are available on request due to restrictions. The data presented in this study are available on request from the corresponding author.

## References

[1] Altaf F., Gibson A., Dannawi Z., Noordeen H. (2013). Adolescent idiopathic scoliosis. BMJ.

[2] Weinstein S.L., Dolan L.A., Cheng J.C., Danielsson A., Morcuende J.A. (2008). Adolescent idiopathic scoliosis. Lancet.

[3] Cheng J.C., Castelein R.M., Chu W.C., Danielsson A.J., Dobbs M.B., Grivas T.B., Gurnett C.A., Luk K.D., Moreau A., Newton P.O. (2015). Adolescent idiopathic scoliosis. Nat. Rev. Dis. Primers.

[4] Lonstein J.E. (2006). Scoliosis: Surgical versus nonsurgical treatment. Clin. Orthop. Relat. Res..

[5] Tones M., Moss N., Polly D.W. (2006). A review of quality of life and psychosocial issues in scoliosis. Spine.

[6] Petcharaporn M., Pawelek J., Bastrom T., Lonner B., Newton P.O. (2007). The relationship between thoracic hyperkyphosis and the Scoliosis Research Society outcomes instrument. Spine.

[7] DiMeglio A., Canavese F., Charles Y.P. (2011). Growth and adolescent idiopathic scoliosis: When and how much?. J. Pediatr. Orthop..

[8] Negrini S., Donzelli S., Aulisa A.G., Czaprowski D., Schreiber S., de Mauroy J.C., Diers H., Grivas T.B., Knott P., Kotwicki T. (2018). 2016 SOSORT guidelines: Orthopaedic and rehabilitation treatment of idiopathic scoliosis during growth. Scoliosis Spinal Disord..

[9] Monticone M., Ambrosini E., Cazzaniga D., Rocca B., Ferrante S. (2014). Active self-correction and task-oriented exercises reduce spinal deformity and improve quality of life in subjects with mild adolescent idiopathic scoliosis. Results of a randomised controlled trial. Eur. Spine J..

[10] Conrad E.J., Becker M., Powell B., Hall K.C. (2020). Improving Health Promotion Through the Integration of Technology, Crowdsourcing, and Social Media. Health Promot. Pract..

[11] Ramanadhan S., Mendez S.R., Rao M., Viswanath K. (2013). Social media use by community-based organizations conducting health promotion: A content analysis. BMC Public Health.

[12] Bose R., Dey R.K., Roy S., Sarddar D. (2019). Analyzing political sentiment using Twitter data. Information and Communication Technology for Intelligent Systems.

[13] Chanda K., Roy S., Mondal H., Bose R. (2022). To Judge Depression and Mental Illness on Social Media Using Twitter. Univers. J. Public Health.

[14] Sarddar D., Dey R.K., Bose R., Roy S. (2020). Topic modeling as a tool to gauge political sentiments from twitter feeds. Int. J. Nat. Comput. Res..

[15] Koetsier J. (2020). Massive TikTok Growth: Up 75% this year, now 33x more users than nearest direct competitor. Retrieved Dec..

[16] Kong W., Song S., Zhao Y.C., Zhu Q., Sha L. (2021). TikTok as a Health Information Source: Assessment of the Quality of Information in Diabetes-Related Videos. J. Med. Internet Res..

[17] Ostrovsky A.M., Chen J.R. (2020). TikTok and Its Role in COVID-19 Information Propagation. J. Adolesc. Health.

[18] Song S., Xue X., Zhao Y.C., Li J., Zhu Q., Zhao M. (2021). Short-Video Apps as a Health Information Source for Chronic Obstructive Pulmonary Disease: Information Quality Assessment of TikTok Videos. J. Med. Internet Res..

[19] Eghtesadi M., Florea A. (2020). Facebook, Instagram, Reddit and TikTok: A proposal for health authorities to integrate popular social media platforms in contingency planning amid a global pandemic outbreak. Can. J. Public Health.

[20] Auxier B., Anderson M. (2021). Social media use in 2021. Pew Res. Cent..

[21] Goobie G.C., Guler S.A., Johannson K.A., Fisher J.H., Ryerson C.J. (2019). YouTube Videos as a Source of Misinformation on Idiopathic Pulmonary Fibrosis. Ann. Am. Thorac. Soc..

[22] Yoo M., Bang M.H., Jang C.W. (2022). Evaluation of YouTube Videos as a Source of Information on Pulmonary Rehabilitation for COPD. Respir Care.

[23] Charnock D., Shepperd S., Needham G., Gann R. (1999). DISCERN: An instrument for judging the quality of written consumer health information on treatment choices. J. Epidemiol Community Health.

[24] Aydin M.F., Aydin M.A. (2020). Quality and reliability of information available on YouTube and Google pertaining gastroesophageal reflux disease. Int. J. Med. Inform..

[25] Olkun H.K., Demirkaya A.A. (2018). Evaluation of Internet Information about Lingual Orthodontics Using DISCERN and JAMA Tools. Turk. J. Orthod..

[26] Bettany-Saltikov J., Parent E., Romano M., Villagrasa M., Negrini S. (2014). Physiotherapeutic scoliosis-specific exercises for adolescents with idiopathic scoliosis. Eur. J. Phys. Rehabil. Med..

[27] Zhang Y., He D., Sang Y. (2013). Facebook as a platform for health information and communication: A case study of a diabetes group. J. Med. Syst..

[28] Rivas J.G., Socarrás M.R., Blanco L.T. (2016). Social Media in Urology: Opportunities, applications, appropriate use and new horizons. Cent Eur. J. Urol..

[29] Cabrera-Maqueda J.M., Minhas J.S. (2018). New Horizons for Stroke Medicine: Understanding the Value of Social Media. Stroke.

[30] Rozenblum R., Bates D.W. (2013). Patient-centred healthcare, social media and the internet: The perfect storm?. BMJ Qual. Saf..

[31] Cordoş A.-A., Bolboacă S.D., Drugan C. (2017). Social media usage for patients and healthcare consumers: A literature review. Publications.

[32] Erdem M.N., Karaca S. (2018). Evaluating the Accuracy and Quality of the Information in Kyphosis Videos Shared on YouTube. Spine.

[33] Staunton P.F., Baker J.F., Green J., Devitt A. (2015). Online Curves: A Quality Analysis of Scoliosis Videos on YouTube. Spine.

